# Supplementary material for: BRCA1-associated structural variations are a consequence of polymerase theta-mediated end-joining
Source: Nat Commun. 2020 Jul 17;11:3615. doi: 10.1038/s41467-020-17455-3 (PMC7368036; doi:10.1038/s41467-020-17455-3)
Supplement: Supplementary file 1 — Supplementary Information [file 41467_2020_17455_MOESM1_ESM.pdf]

Supplementary Information

BRCA1-associated structural variations are a consequence of polymerase Theta-Mediated End-Joining

Kamp et al.

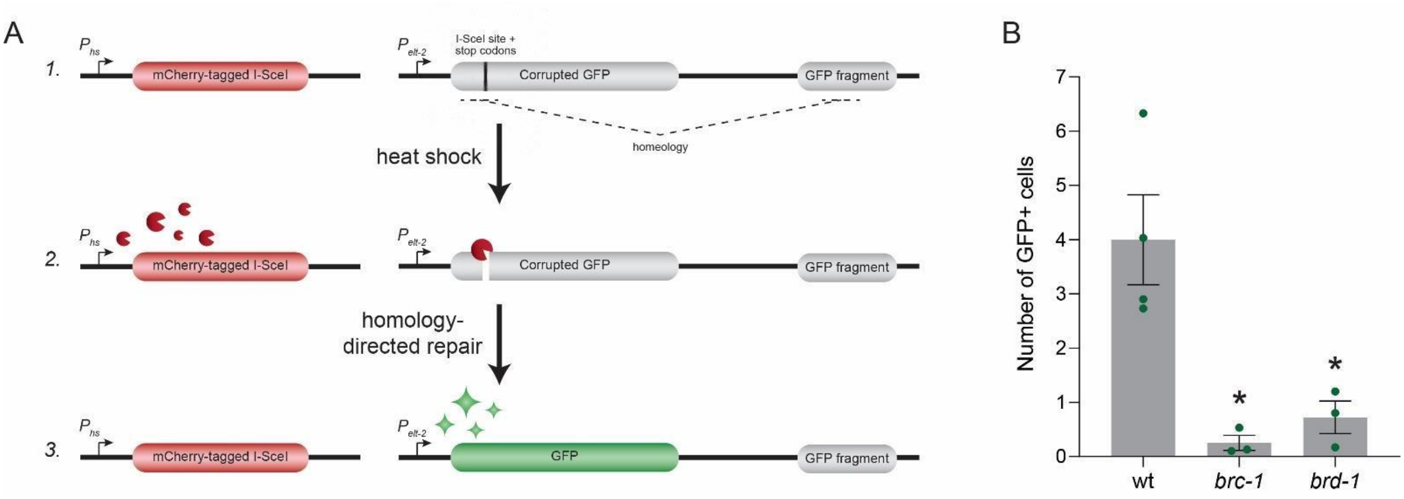

Supplementary figure 1 Homology-directed repair assay (a) Schematic representation of the homology driven repair(HDR) GFP reporter assay described previously[1]. A mCherry-tagged I-SceI endonuclease is expressed after applying a heat shock. The I-SceI generates a double strand break in a corrupted GFP sequence, of which the reading frame can be restored via homology-directed repair of a donor GFP sequence (b) Mean number of GFP+ cells for wild-type (n=4), *brc-1* (n=3**)** and *brd1* (n=3) reporter animals three days after heat shock (two-tailed t-tests vs. wt: *brc-1*: p=0.0128, *brd-1*: p=0.0231). *=P<0.05. Error bars depict SEM.

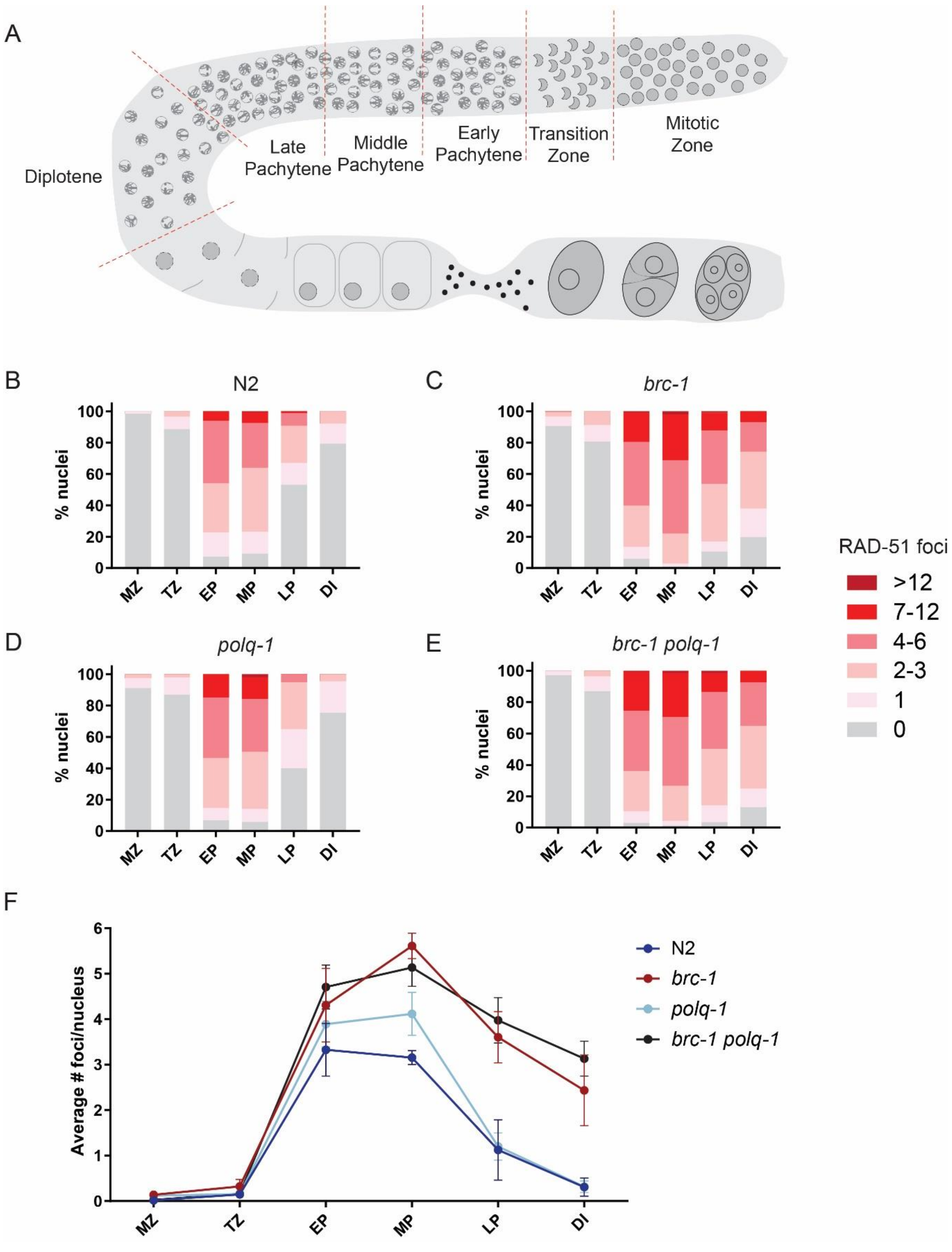

Supplementary figure 2 (a) Schematic representation of a single *C. elegans* gonadal arm. During scoring of RAD-51 foci, germlines were divided according to the indicated zones. (b-e) Bar graphs depicting RAD-51 foci scores for N2, *brc-1*, *polq-1* and *brc-1 polq-1* animals, respectively. Acronyms indicated on the x-axis correspond with the germline zones given in (a). Values are included as a percentage of total nuclei. (f) average number of RAD-51 foci per zone. Three germlines were scored per genotype. Error bars depict SEM.

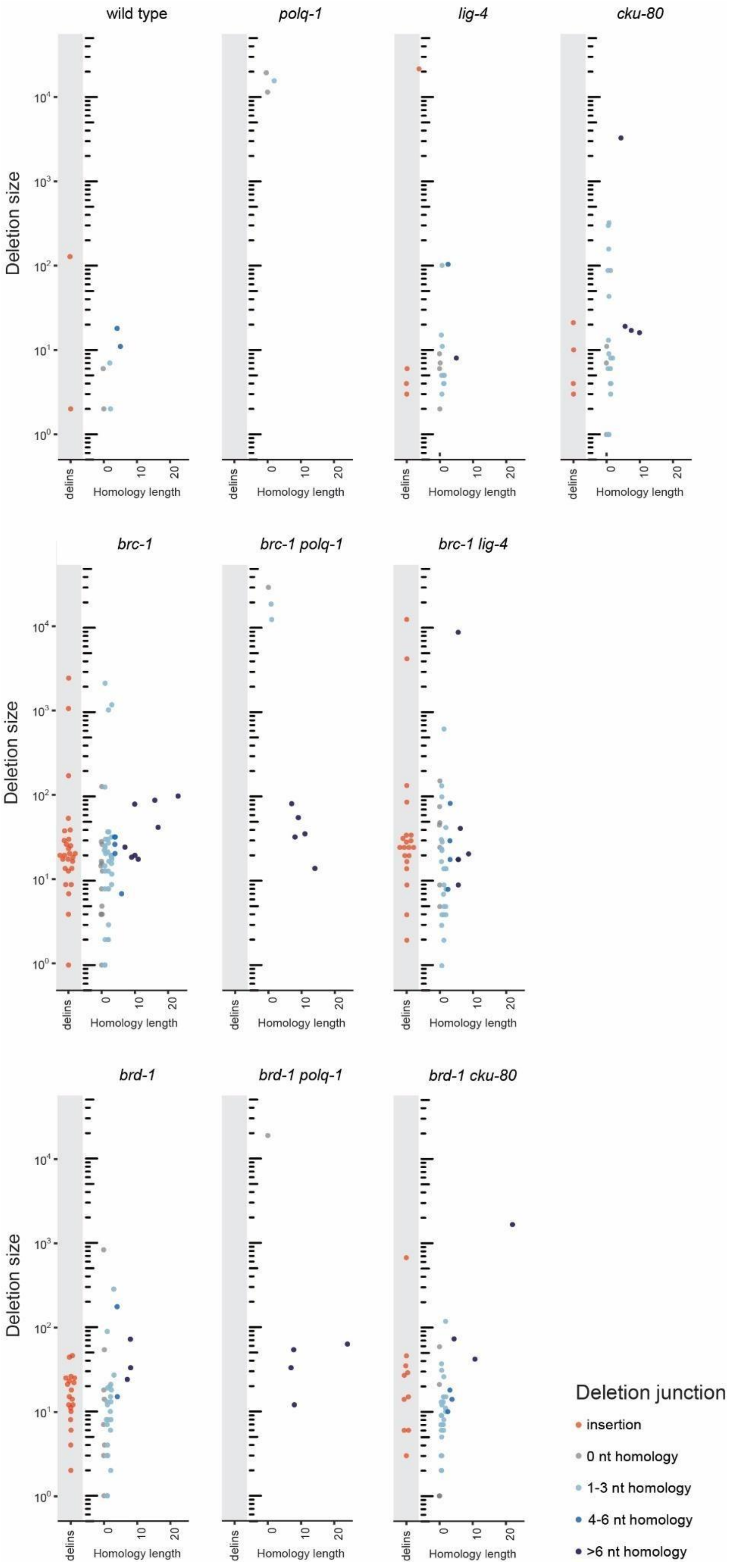

wild type
polq-1
lig-4
cku-80
brc-1
brc-1 polq-1
brc-1 lig-4
brd-1
brd-1 polq-1
brd-1 cku-80
Deletion size
10^0
10^1
10^2
10^3
10^4
delins
Homology length
0
10
20
Deletion junction
insertion
0 nt homology
1-3 nt homology
4-6 nt homology
>6 nt homology

Supplementary figure 3 Deletions plotted sorted on amount of homology (x-axis). Deletions without homology are marked in grey, deletions with homology are marked in blue. Increasing homology size is depicted by increased colour intensity. Deletions with insertions are marked in red.

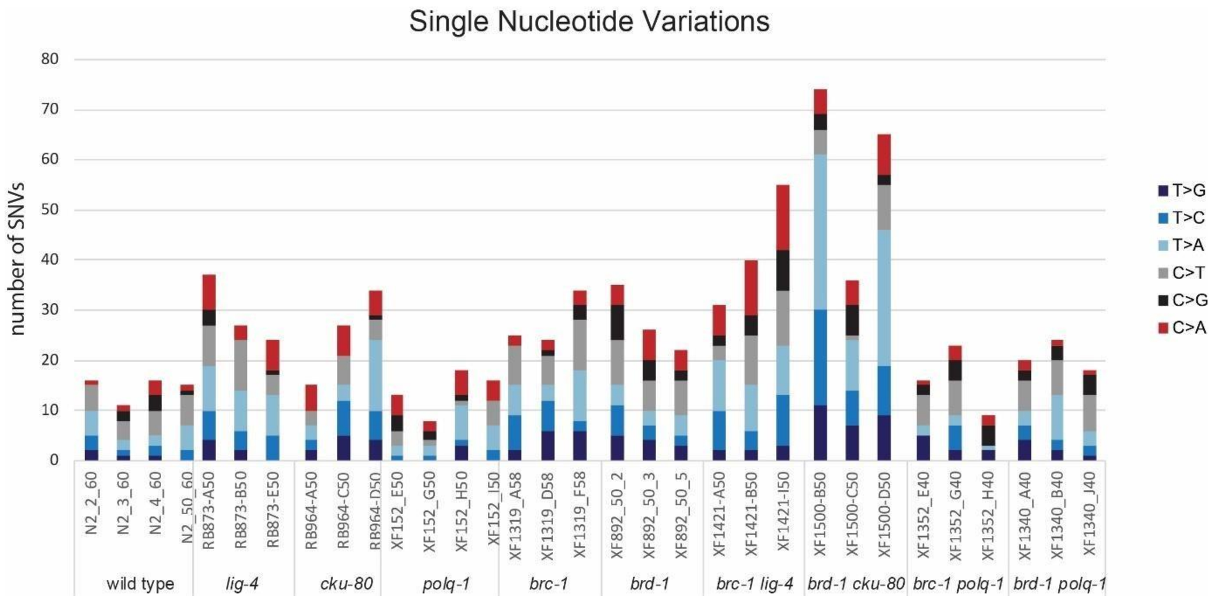

Supplementary figure 4 Single nucleotide variants per sample. The different types of base substitutions are labelled with different colours.

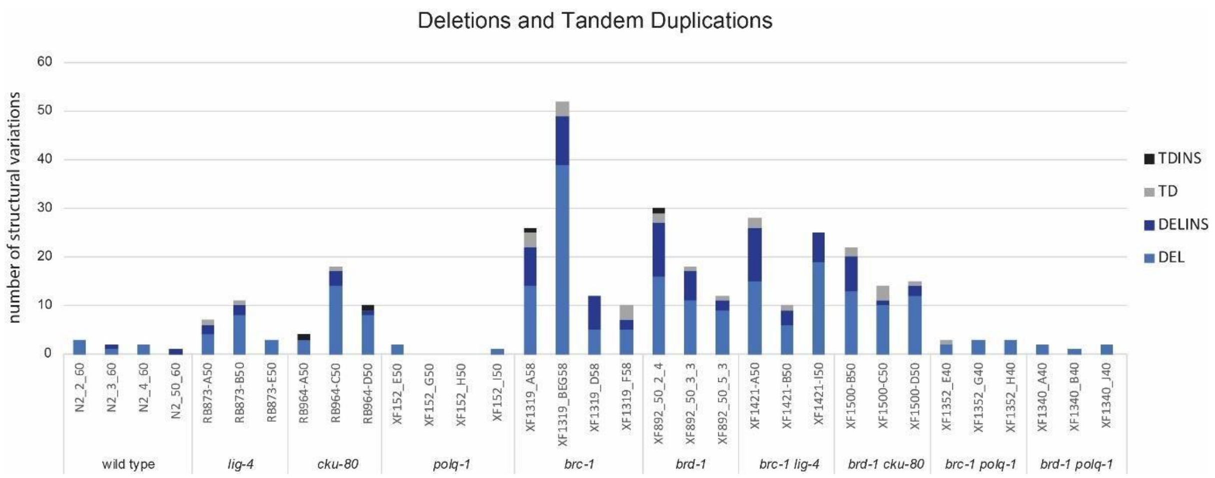

Supplementary figure 5 Deletions and tandem duplications per sample. The different types of variations are labelled with different colours.

Gene	Allele	Left flank	Right flank	Deletion	Insertion
brc-1	*lf249*	GTTGCACTGAGG	ACTGCAAAAAGA	ATCACAGAAACAGTGGCACG	TTTCTT
polq-1	*lf265*	AGAACATCAAAG	ACAAGTGCTGGG	CGCTGTTCGATTGGCAGATTGATGTG TTGAATGAGGCAAGACAATTTGAAGA TCAACACTTGATATTCAGTGCGCCA	-
polq-1	*lf257*	TTGATGTGTTGA	ATGAGGCAAGAC	-	TGTGTTGATTG

Supplementary table 1. Novel alleles made for this study. Deletions and insertions induced by CRISPR/Cas9 are described and the 12 nucleotides left and right of the event are indicated.

FILE	AVERAGE COVERAGE
**N2_2_60.SORTED.BAM**	36,7043
**N2_3_60.SORTED.BAM**	60,865
**N2_4_60.SORTED.BAM**	19,7175
**N2_50_60.SORTED.BAM**	27,6871
**RB873-A50.SORTED.BAM**	52,9499
**RB873-B50.SORTED.BAM**	50,101
**RB873-E50.SORTED.BAM**	60,5659
**RB964-A50.SORTED.BAM**	90,4804
**RB964-C50.SORTED.BAM**	49,4818
**RB964-D50.SORTED.BAM**	50,2128
**XF1319-A58.SORTED.BAM**	45,4568
**XF1319-BEG58.SORTED.BAM**	43,6232
**XF1319-D58.SORTED.BAM**	49,5294
**XF1319-F58.SORTED.BAM**	25,9137
**XF1340-A40.SORTED.BAM**	41,6894
**XF1340-B40.SORTED.BAM**	42,1044
**XF1340-J40.SORTED.BAM**	45,6015
**XF1352-E40.SORTED.BAM**	36,3602
**XF1352-G40.SORTED.BAM**	30,2931
**XF1352-H40.SORTED.BAM**	40,759
**XF1421-0.SORTED.BAM**	32,1812
**XF1421-A50.SORTED.BAM**	50,3571
**XF1421-B50.SORTED.BAM**	62,1379
**XF1421-I50.SORTED.BAM**	41,4118
**XF1500-0.SORTED.BAM**	47,4243
**XF1500-B50.SORTED.BAM**	54,4259
**XF1500-C50.SORTED.BAM**	72,7008

**XF1500-D50.SORTED.BAM**	69,3614
**XF152_E50.SORTED.BAM**	42,6985
**XF152_G50.SORTED.BAM**	19,2885
**XF152_H50.SORTED.BAM**	38,1669
**XF152_I50.SORTED.BAM**	43,1527
**XF892_50_2_4.SORTED.BAM**	25,8014
**XF892_50_3_3.SORTED.BAM**	26,8575
**XF892_50_5_3.SORTED.BAM**	20,1802

Supplementary table 2 Average coverage per sample. N2= wild type, RB873=*lig-4,* R964=*cku-80,* XF1319=*brc-1,* XF1340=*brd-1 polq-1,* XF1352=*brc-1 polq-1,* XF1421=*brc-1 lig-4,* XF1500=*brd-1 cku-80,* XF152= *polq-1,* XF892=*brd-1*

Sample	Strain	Genotype	Generations
**XF1319_A58**	XF1319	*brc-1*	58
**XF1319_BEG58**	XF1319	*brc-1*	3x58
**XF1319_D58**	XF1319	*brc-1*	58
**XF1319_F58**	XF1319	*brc-1*	58
**XF1421-0**	XF1421	*brc-1 lig-4*	0
**XF1421-B50**	XF1421	*brc-1 lig-4*	50
**XF1421-A50**	XF1421	*brc-1 lig-4*	50
**XF1421-I50**	XF1421	*brc-1 lig-4*	50
**XF1352_E40**	XF1352	*brc-1 polq-1*	40
**XF1352_G40**	XF1352	*brc-1 polq-1*	40
**XF1352_H40**	XF1352	*brc-1 polq-1*	40
**XF892_50_2_4**	XF892	*brd-1*	50
**XF892_50_3_3**	XF892	*brd-1*	50
**XF892_50_5_3**	XF892	*brd-1*	50
**XF1500-0**	XF1500	*brd-1 cku-80*	0
**XF1500-D50**	XF1500	*brd-1 cku-80*	50
**XF1500-B50**	XF1500	*brd-1 cku-80*	50
**XF1500-C50**	XF1500	*brd-1 cku-80*	50
**XF1340_A40**	XF1340	*brd-1 polq-1*	40
**XF1340_B40**	XF1340	*brd-1 polq-1*	40
**XF1340_J40**	XF1340	*brd-1 polq-1*	40
**RB964-C50**	RB964	*cku-80*	50
**RB964-D50**	RB964	*cku-80*	50
**RB964-A50**	RB964	*cku-80*	50
**RB873-B50**	RB873	*lig-4*	50
**RB873-A50**	RB873	*lig-4*	50
**RB873-E50**	RB873	*lig-4*	50
**XF152_E50**	XF152	*polq-1*	50
**XF152_G50**	XF152	*polq-1*	50
**XF152_H50**	XF152	*polq-1*	50
**XF152_I50**	XF152	*polq-1*	50

**N2_2_60**	N2	*WT*	60
**N2_3_60**	N2	*WT*	60
**N2_4_60**	N2	*WT*	60
**N2_50_60**	N2	*WT*	60

Supplementary table 3 Number of generations per sample All samples besides XF1319_BEG contain DNA of one mutation accumulation line, besides XF1319_BEG58, which contains the DNA of three mutation accumulation lines.

Supplementary References

1 Johnson, N. M., Lemmens, B. B. & Tijsterman, M. A role for the malignant brain tumour (MBT) domain protein LIN-61 in DNA double-strand break repair by homologous recombination. *PLoS Genet* **9**, e1003339 (2013).
