# Supplementary material for: BRCA1-associated structural variations are a consequence of polymerase theta-mediated end-joining
Source: Nat Commun. 2020 Jul 17;11:3615. doi: 10.1038/s41467-020-17455-3 (PMC7368036; doi:10.1038/s41467-020-17455-3)
Supplement: Supplementary file 3 — Description of Additional Supplementary Files [file 41467_2020_17455_MOESM3_ESM.pdf]

Description of Additional Supplementary Files

File Name: Supplementary Data 1
Description: **Table of mutations described in this study.** In the 'Structural variations' sheet, location and size of all deletions and tandem duplications depicted in the figures are listed. When applicable, the nature and length of homology at the break site is reported. When the structural variations contain an insertion, the insertion is described in the 'Insert' column. The 'FlankInsert' column reports whether the insert could be originating from the sequence flanking the structural variation. In the 'FlankInsertLargestMatch' column, the largest string of insert nucleotides that match with the flank is included. In the 'SNVs' sheet, all base substitutions depicted in the figures are listed. The reference nucleotide and the alternative nucleotide are mentioned in the 'Ref' and 'Alt' columns. The mutation is described in the 'Mutation' column.

File Name: Supplementary Data 2
Description: **Data corresponding to figure 4**. The average percentage of alive progeny per replicate experiment is included in this document
