# Supplementary material for: BRCA1-associated structural variations are a consequence of polymerase theta-mediated end-joining
Source: Nat Commun. 2020 Jul 17;11:3615. doi: 10.1038/s41467-020-17455-3 (PMC7368036; doi:10.1038/s41467-020-17455-3)
Supplement: Supplementary file 6 — Reporting Summary [file 41467_2020_17455_MOESM6_ESM.pdf]

nature research

Corresponding author(s): Marcel Tijsterman

Last updated by author(s): Jun 16, 2020

Reporting Summary

Nature Research wishes to improve the reproducibility of the work that we publish. This form provides structure for consistency and transparency in reporting. For further information on Nature Research policies, see our Editorial Policies and the Editorial Policy Checklist.

Statistics

For all statistical analyses, confirm that the following items are present in the figure legend, table legend, main text, or Methods section.

n/a	Confirmed	
☐	☒	The exact sample size (*n*) for each experimental group/condition, given as a discrete number and unit of measurement
☒	☐	A statement on whether measurements were taken from distinct samples or whether the same sample was measured repeatedly
☐	☒	The statistical test(s) used AND whether they are one- or two-sided<br>*Only common tests should be described solely by name; describe more complex techniques in the Methods section.*
☒	☐	A description of all covariates tested
☒	☐	A description of any assumptions or corrections, such as tests of normality and adjustment for multiple comparisons
☐	☒	A full description of the statistical parameters including central tendency (e.g. means) or other basic estimates (e.g. regression coefficient) AND variation (e.g. standard deviation) or associated estimates of uncertainty (e.g. confidence intervals)
☐	☒	For null hypothesis testing, the test statistic (e.g. *F*, *t*, *r*) with confidence intervals, effect sizes, degrees of freedom and *P* value noted<br>*Give P values as exact values whenever suitable.*
☒	☐	For Bayesian analysis, information on the choice of priors and Markov chain Monte Carlo settings
☒	☐	For hierarchical and complex designs, identification of the appropriate level for tests and full reporting of outcomes
☒	☐	Estimates of effect sizes (e.g. Cohen's *d*, Pearson's *r*), indicating how they were calculated

Our web collection on statistics for biologists contains articles on many of the points above.

Software and code

Policy information about availability of computer code

Data collection	Image analysis, base calling and error calibration were performed using standard Illumina software: BCL output from the HiSeqX and Novaseq6000 platform was converted using bcl2fastq tool (Illumina, versions 2.17 to 2.20 have been used) using default parameters. Raw reads were mapped to the C. elegans reference genome (Wormbase release 235) by BWA (version 0.7.17) and further processed via SAMtools(1.10).
Data analysis	Pindel (0.2.5a8), GATK(4.0.8.1), Manta(1.6.0) and GRIDSS(2.8.0) were used for callling structural variations, GATK(4.0.8.1) was used for SNV calling. Mutations were visually inspected using IGV (2.8.3)

For manuscripts utilizing custom algorithms or software that are central to the research but not yet described in published literature, software must be made available to editors and reviewers. We strongly encourage code deposition in a community repository (e.g. GitHub). See the Nature Research guidelines for submitting code & software for further information.

Data

Policy information about availability of data

All manuscripts must include a data availability statement. This statement should provide the following information, where applicable:

- Accession codes, unique identifiers, or web links for publicly available datasets
- A list of figures that have associated raw data
- A description of any restrictions on data availability

Raw sequences have been made publicly available at NCBI SRA (Accession code PRJNA599297). Data for N2 wild-type and polq-1 animals were published previously and can be found at NCBI SRA (Accession codes PRJNA260487 and PRJNA196232). A list of all events and raw data of figure 4 are available as supplementary data. No unreported custom computer code was used during this study. Database used: C. elegans reference genome (Wormbase -release 235).

Field-specific reporting

Please select the one below that is the best fit for your research. If you are not sure, read the appropriate sections before making your selection.

☒ Life sciences ☐ Behavioural & social sciences ☐ Ecological, evolutionary & environmental sciences

For a reference copy of the document with all sections, see nature.com/documents/nr-reporting-summary-flat.pdf

Life sciences study design

All studies must disclose on these points even when the disclosure is negative.

Sample size	For NGS experiments we found in previous publications that ~3*50 generations/genotype provides a sample size sufficient to analyze in case mutants have a different mutation signature (see: Van Schendel et al., Nat. Commun. 2015, DOI: 10.1038/ncomms8394 & Van Bostelen et al., PloS Genet 2020, DOI: 10.1371/journal.pgen.1008759), therefore we choose 3 lines as a minimum to sequence.<br><br>For the IR experiment, the required sample size is based on literature and previous work (e.g. Johnson et al, Plos Genet. 2012, DOI: 10.1371/journal.pgen.1003339). The same goes for germline stainings: three germlines per genotype is the standard in C. elegans literature and we performed similar experiments previously (Lemmens et al., Plos Genet.2013, DOI: 10.1371/journal.pgen.1003276). The sample sizes for the supplemental GFP reporter data was also based on previous work (Johnson et al, Plos Genet. 2012, DOI: 10.1371/journal.pgen.1003339).
Data exclusions	No data was excluded.
Replication	Apart from the usual performing experiments in multiplo, if possible, we used both brc-1 and brd-1 alleles in the experiments, which functionally require each other, to ensure reproducibility. No differences were observed, as depicted in figure 1 in the manuscript. We also performed the IR experiment with brd-1 and brd-1 polq-1 strains instead of brc-1 and brc-1 polq-1 and we obtained similar results. We also performed an IR experiment with another brc-1 polq-1 strain, in which the brc-1 allele was another null allele than used in this study. Again, we found similar results.
Randomization	Allocation was random: For each experiment, the first worm (in the right developmental stage for the experiment) that was seen through the microscope when the stock plate was observed was picked precluding potential bias introduced by the experimentator
Blinding	Some experiments were blinded: the genotype and dose (if applicable) was not known when scoring the plates/microscopy slides in the IR experiment (fig 4), HDR reporter experiment (fig S1) and RAD-51 staining (fig S2).<br>The mutation accumulation line experiment was not blinded, since the events were called by software and not scored by humans.

Reporting for specific materials, systems and methods

We require information from authors about some types of materials, experimental systems and methods used in many studies. Here, indicate whether each material, system or method listed is relevant to your study. If you are not sure if a list item applies to your research, read the appropriate section before selecting a response.

Materials & experimental systems

n/a	Involved in the study	
☐	☒	Antibodies
☒	☐	Eukaryotic cell lines
☒	☐	Palaeontology and archaeology
☐	☒	Animals and other organisms
☒	☐	Human research participants
☒	☐	Clinical data
☒	☐	Dual use research of concern

Methods

n/a	Involved in the study	
☒	☐	ChIP-seq
☒	☐	Flow cytometry
☒	☐	MRI-based neuroimaging

Antibodies

Antibodies used	RAD51 antibody (Novus biologicals) and secondary Alexa488-labeled goat-anti-rabbit antibody (Invitrogen).<br>Secondary Alexa488-labeled goat-anti-rabbit antibody (Invitrogen).
Validation	"C. elegans reactivity reported in scientific literature (PMID: 23942865)" (source: https://www.novusbio.com/primaryantibodies/rad51)

Animals and other organisms

Policy information about studies involving animals; ARRIVE guidelines recommended for reporting animal research

Laboratory animals	Caenorhabditis elegans, hermaphrodites, L1-adult stage, strains: N2, RB873, R964, XF1319, XF1340, XF1352, XF1421, XF1500, XF152 and XF892
Wild animals	The study did not involve wild animals
Field-collected samples	The study did not involve field-collected samples
Ethics oversight	No ethical approval was required, since C.elegans is an invertebrate

Note that full information on the approval of the study protocol must also be provided in the manuscript.
